# System Design and Echo Preprocessing of Spaceborne Squinted Two-Dimensional Beam Scanning Synthetic Aperture Radar

**DOI:** 10.3390/s23208377

**Published:** 2023-10-10

**Authors:** Wei Xu, Xuhang Lu, Pingping Huang, Weixian Tan, Zhiqi Gao, Yaolong Qi

**Affiliations:** 1 College of Information Engineering, Inner Mongolia University of Technology, Hohhot 010051, China; 20211800129@imut.edu.cn (X.L.); hwangpp@imut.edu.cn (P.H.); wxtan@imut.edu.cn (W.T.); gzqngd@imut.edu.cn (Z.G.); qiyaolong@imut.edu.cn (Y.Q.); 2 Inner Mongolia Key Laboratory of Radar Technology and Application, Hohhot 010051, China

**Keywords:** synthetic aperture radar (SAR), squinted sliding spotlight, two-dimensional (2D) beam scanning, high resolution wide swath (HRWS), SAR imaging

## Abstract

Conventional squinted sliding spotlight synthetic aperture radar (SAR) imaging suffers from substantial swath width reduction and complex processing requirements due to the continuous variation in the squint angle and the large range cell migration (RCM) throughout the data acquisition interval. A novel two-dimensional (2D) beam scanning mode for high-resolution wide swath (HRWS) imaging is proposed. The key to the novel imaging mode lies in the synchronous scanning of azimuth and range beams, allowing for a broader and more flexible imaging swath with a high geometric resolution. Azimuth beam scanning from fore to aft was used to improve the azimuth resolution, while range beam scanning was adopted to illuminate the oblique wide swath to avoid the large RCM and the serious swath width reduction. Compared with the conventional sliding spotlight mode, both the swath width and swath length could be extended. According to the echo model of this imaging mode, an echo signal preprocessing approach is proposed. The key points of this approach are range data extension and azimuth data upsampling. A designed system example with a resolution of 0.5 m, swath width of 60 km, and azimuth coverage length of 134 km is presented. Furthermore, a simulation experiment on point targets was carried out. Both the presented system example and imaging results of point targets validated the proposed imaging mode.

## 1. Introduction

Spaceborne synthetic aperture radar (SAR) systems play a vital role in tasks like regional mapping, geophysical parameter inversion, and disaster assessment thanks to their all-weather, day-and-night, long-endurance imaging capabilities with fine resolutions [1,2,3,4,5]. In the sliding spotlight SAR mode, the azimuth resolution is improved using azimuth beam scanning, which enlarges the Doppler bandwidth [6,7]. The adjustable squint angle in the squinted spotlight mode provides more flexibility in multi-angle observations and rapid target revisits within one orbital pass [8,9,10,11]. Moreover, the coherent processing of echoed data from both the forward and backward squinted beams enables an enlarged equivalent antenna aperture for a higher signal-to-noise ratio [12,13,14,15]. Spaceborne squinted sliding spotlight SAR has provided a significant imaging mode that has found extensive use in applications like disaster monitoring and environmental surveying, which is attributable to its advantages of high signal-to-noise ratio, high radiometric resolution, and variable squint angle. However, the practical implementation of squinted sliding spotlight SAR faces two primary challenges that can undermine the system’s performance. First, the increased scanning angle under the large squint case will lead to pulse overlap between transmission and reception due to the large range cell migration (RCM), resulting in an obviously reduced swath. Second, the insufficient pulse repetition frequency (PRF) of squinted systems combined with the conventional two-step processing approach will induce Doppler spectrum aliasing.

Many scholars have conducted extensive studies on the two issues mentioned. First, the PRF is continuously changed during the whole raw data acquisition interval to avoid the swath reduction due to the large RCM. However, this method leads to a significant increase in the variable ratio of the PRF, which can adversely affect the system’s performance by causing severe non-uniformity in the PRF. While some researchers employed a block-variable PRF method to address challenges in the squinted sliding spotlight mode [16], this approach leads to the emergence of false targets. The continuous varying pulse interval method was proposed to simultaneously avoid transmission blockage and maximize the allowable width of the region of interest [17,18]. Second, the PRF can be reduced by employing classical HRWS multi-channel SAR imaging techniques, such as displaced phase multiple azimuth beams (DPCMABs), single phase multiple azimuth beams (SPMABs), multiple elevation beams (MEBs), and elevation digital beamforming (DBF) [19,20,21,22]. However, the implementation of these multi-channel techniques necessitates an increase in antenna length and the number of channels, thereby adding complexity to the system.

To extend the swath width of the squinted sliding spotlight mode, a novel two-dimensional (2D) beam scanning mode for a high-resolution wide swath (HRWS) is proposed in this paper. In contrast with the traditional squinted sliding spotlight mode, which solely scans in the azimuth direction, the proposed imaging mode in this paper allows for antenna beam scanning in terms of both the azimuth and elevation. Additionally, this mode can leverage coherent information from various angles to mitigate interference and noise, subsequently improving the imaging quality and resolution. By varying the range-oriented scanning angle, the echo window remains relatively stable, allowing for efficient utilization of the echo window without the need to adjust the PRF. This approach enables the acquisition of a wider swath width without adding to the system load, ultimately yielding larger imaging areas and higher-resolution imaging outcomes. Notably, this mode maintains a roughly constant equivalent nadir angle, ensuring effective swath acquisition and significantly suppressing the range migration, even as the squint scanning angle increases. Furthermore, this mode accommodates targets that are not parallel to the satellite ground track, enhancing the system’s adaptability and performance. According to echo data characteristics, an echo preprocessing approach is proposed to handle the raw data of the proposed novel imaging mode. The key points of the proposed echo preprocessing approach are range data extension and azimuth data upsampling. Range data extension was introduced to avoid the final focused SAR image folding due to the oblique imaged scene, while azimuth data upsampling was adopted to resolve the aliased Doppler spectrum due to the large azimuth scanning angle under the high squint angle case. After echo preprocessing, the resulting raw data can be handled by the classical spaceborne high-resolution imaging processors [23].

The organization of this article unfolds as follows. Section 2 addresses the challenges inherent in the conventional squinted sliding spotlight mode, highlighting the need for an innovative imaging mode for HRWS imaging. In Section 3, a novel squinted 2D beam scanning SAR imaging mode is proposed, offering a fresh perspective on the issue. Its corresponding echo data preprocessing approach is presented, which is operated before the classical SAR imaging processors. In Section 4, the designed imaging mode examples are given. Additionally, a simulation experiment on point targets is carried out in Section 5 to validate the proposed imaging mode. Finally, this paper is concluded in Section 6.

## 2. Challenges in Traditional Squinted Sliding Spotlight Mode

**Figure 1:**
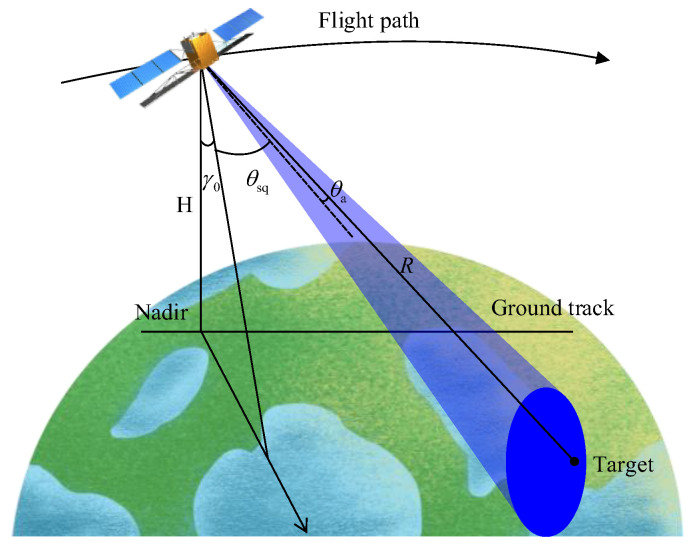
Geometries of traditional squinted spaceborne SAR.

The squinted mode has broad applicability but faces constraints regarding resolution and coverage. In conventional mode, there is no effective azimuth synthetic aperture, as shown in Figure 1. This results in a lower azimuth resolution and a smaller swath width. This mode also increases the range migration complexity. Moreover, the migration may exceed the echo window size, limiting the ground coverage and continuous large-area acquisition. Due to the narrow swath, the traditional mode requires multiple overflights for large areas, which is time-consuming and costly.

The expression for the instantaneous squint distance $R(\gamma ,\theta_{\mathrm{sq}};\theta_{a})$ with respect to a given target can be derived based on Figure 1 as follows:

(1) $$R(\gamma ,\theta_{\mathrm{sq}};\theta_{a})=\sqrt{{\left(R_{e}+H\right)}^{2}+R_{e}{}^{2}-2(R_{e}+H)R_{e}\cos\beta (t)}$$

with

(2) $$\beta (\gamma ,\theta_{\mathrm{sq}};\theta_{a})=\alpha (\gamma ,\theta_{\mathrm{sq}};\theta_{a})+\theta_{a}-\gamma_{\mathrm{eq}}(\gamma ,\theta_{\mathrm{sq}};\theta_{a})$$

(3) $$\alpha (\gamma ,\theta_{\mathrm{sq}};\theta_{a})=\sin^{-1}\left[\frac{R_{e}+H}{R_{e}}\sin\gamma_{\mathrm{eq}}(\gamma ,\theta_{\mathrm{sq}};\theta_{a})\right]$$

(4) $$\gamma_{\mathrm{eq}}(\gamma ,\theta_{\mathrm{sq}};\theta_{a})=\cos^{-1}\left[\cos\gamma \cos\left(\theta_{\mathrm{sq}}(t)+\theta_{a}(t)\right)\right]$$

where $\beta (\gamma ,\theta_{\mathrm{sq}};\theta_{a})$ is the geocentric angle; γ(t) is the looking angle; $\alpha (\gamma ,\theta_{\mathrm{sq}};\theta_{a})$ is the incident angle; $\gamma_{\mathrm{eq}}(\gamma ,\theta_{\mathrm{sq}};\theta_{a})$ is the nadir angle; H is the orbital height; and $\theta_{a}$ is the exploited azimuth beam interval, which is defined as the angle deviation from the beam center, i.e., $\theta_{a}\in \left[-\theta /2,\theta /2\right)$.

The duration of the echo across the entire intended range swath can be formulated as follows, where $\tau_{p}$ signifies the transmitted pulse’s duration:

(5) $$T_{\mathrm{echo}}\left(\theta_{\mathrm{sq}}\right)=\frac{2}{c}\left[R\left(\gamma_{\mathrm{far}},\theta_{\mathrm{sq}}(t);\theta_{a}/2\right)-R\left(\gamma_{\mathrm{near}},\theta_{\mathrm{sq}}(t);-\theta_{a}/2\right)\right]+\tau_{p}$$

The echo duration throughout the azimuth acquisition interval can be calculated as follows:

(6) $$\begin{array}{ll} T_{\mathrm{total}}(\theta_{\mathrm{sq}}) & =\frac{2}{c}\left[\max\left(R(\gamma ,\theta_{\mathrm{sq}}(t);\theta_{\mathrm{az}})\right)-\min\left(R(\gamma ,\theta_{\mathrm{sq}}(t);\theta_{\mathrm{az}})\right)\right]+\tau_{p}\\ & =\frac{2}{c}\left[R(\gamma_{\mathrm{far}},\theta_{a}-\omega_{r}T/2;\theta_{\mathrm{az}})-R(\gamma_{\mathrm{near}},\theta_{a}+\omega_{r}T/2;\theta_{\mathrm{az}})\right]+\tau_{p} \end{array}$$

Let $r_{\mathrm{near}}$ and $r_{\mathrm{far}}$ denote the near and far slant ranges of the beam; then, the PRI change range preventing pulse transmission from erroneously entering the reception window is

(7) $$\frac{2r_{\mathrm{far}}(t)/c+\tau_{\mathrm{pro}}}{nss+1}\leq \mathrm{PRI}\leq \frac{2r_{\mathrm{far}}(t)/c-\tau_{p}-\tau_{\mathrm{pro}}}{nss}$$

The range migration within the observation area increases with squint angle and can be calculated as follows:

(8) $$\Delta R_{\mathrm{rcm}}(t)=r_{\mathrm{far}}(t)-\min\left(r_{\mathrm{near}}(t)\right)$$

**Figure 2:**
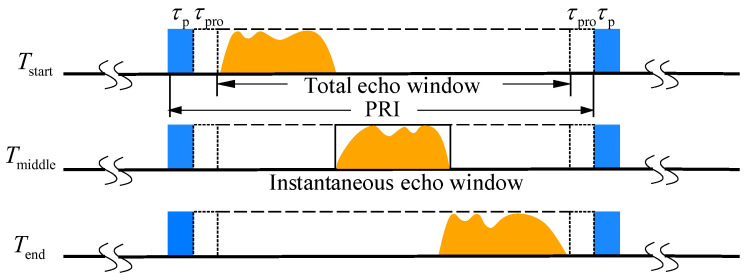
The variation in the traditional squinted sliding spotlight echo window over time.

Receiving echo signals presents challenges, as shown in Figure 2. Due to constraints, echoes may split into separate windows, which need specialized processing for seamless observation. This splitting decreases the echo proportion within the window, reducing the data usage. Also, the fluctuating radar–target distance shifts the echo’s range position, necessitating a range cell migration correction. Together, these factors contribute to unstable radar data reception and poorer image quality.

$\Gamma_{1}$ is defined as the proportion of the echo ratio, which is expressed as follows:

(9) $$\Gamma_{1}=T_{\mathrm{echo}}(\theta_{\mathrm{sq}})/\mathrm{PRI}$$

$\Gamma_{2}$ is defined as the proportion of the PRI, which is expressed as follows:

(10) $$\Gamma_{2}=T_{\mathrm{total}}(\theta_{\mathrm{sq}})/\mathrm{PRI}$$

**Figure 3:**
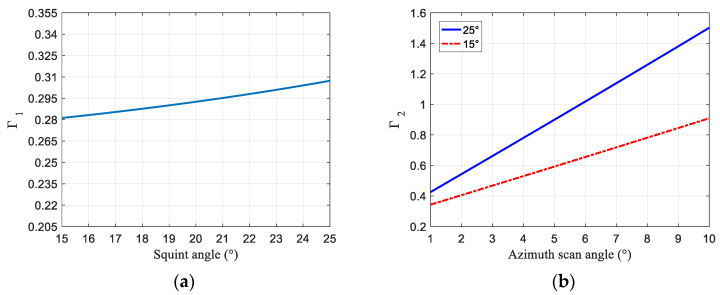
Echo proportion within PRI versus squint angle and azimuth scanning angle. (**a**) Echo proportion within PRI. (**b**) Echo proportion within PRI under varying azimuth scanning angles.

Figure 3a shows the linear relationship between the squint angle and the echo ratio in blue. The echo ratio remains stable from 15 to 25 degrees, confirming that the squint angle adjustment has negligible influence. Figure 3b reveals the significant echo ratio changes with the change in scanning angle. At 25 degrees, higher ratios occur compared with 15 degrees, as shown in blue and red. Given a PRF of 3000 Hz, the pulse time is 12% of the echo window, leaving 88% available. Thus, data loss can occur if the echo ratio exceeds 0.88, worsening with larger squint angles and a PRI that increase redundancy.

**Figure 4:**
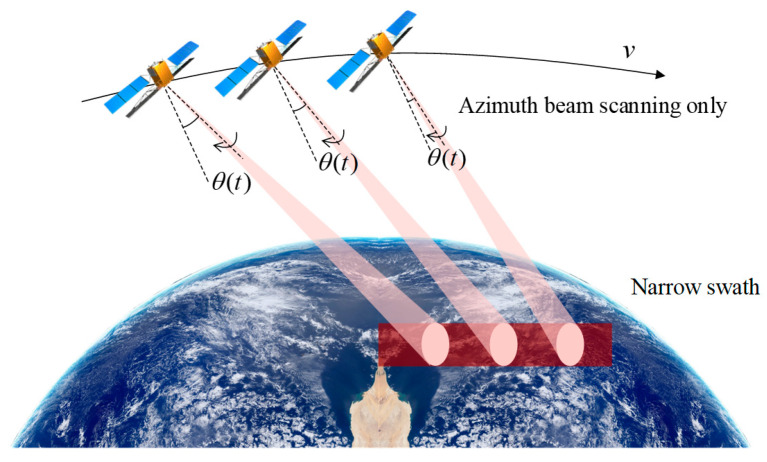
Schematic diagram of slant range in squinted sliding spotlight mode.

**Figure 5:**
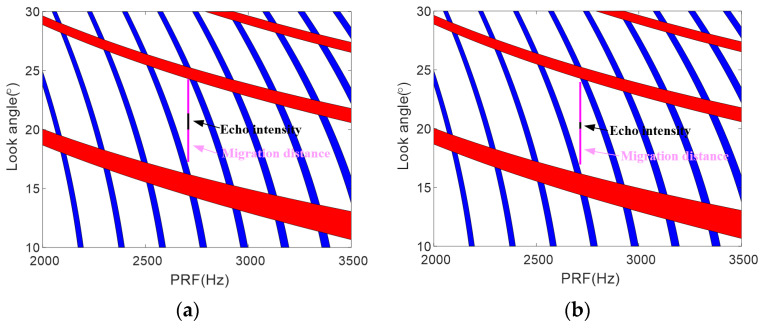
Timing diagram of near-range migration with 15° central squint. (**a**) A ±4° squint angle variation maintains 20 km swath width. (**b**) A ±4.5° squint angle variation maintains 8 km swath width.

Figure 4 aims to underscore the notable reduction in swath width due to variations in squint angles in traditional squinted SAR systems. As depicted in Figure 5a, where the blue and red areas represent the interference regions caused by the lowest point echoes and transmitted pulses, with a ±4° squint angle variation around the central 15° squint, the swath width measures approximately 20 km. However, when the squint angle range expands to ±4.5°, as illustrated in Figure 5b, the swath width diminishes to around 8 km. This reduction is attributed to the increased range cell migration caused by the expanded squint angle range, which, in turn, diminishes the effective swath width that can be imaged without aliasing. Specifically, this emphasizes that a minor 0.5° increment in the squint angle scan range leads to a considerable increase in range cell migration, resulting in a significant contraction of the usable imaging swath width from 20 km to 8 km. This serves to accentuate the inherent limitations in swath coverage of traditional squinted SAR systems due to increased range cell migration with larger squint angles.

To address the reduced swath width, adjusting the PRF is viable but may cause non-uniform azimuth sampling, which requires extensive interpolation. Alternatively, multi-channel techniques receive simultaneous echoes from different beam angles, effectively expanding the relatively small mapping swath of the conventional squinted sliding spotlight mode. However, limitations exist. Various processing methods are available, each with drawbacks. Thus, applications require weighing the pros and cons of each method for optimal imaging.

## 3. Two-Dimensional Beam Scanning SAR for High-Resolution Wide-Swath Imaging

### 3.1. Two-Dimensional Beam Scanning SAR

To tackle the limitations in swath width, resolution, and processing of traditional squinted sliding spotlight SAR, researchers have explored methods like a variable PRF, multi-scale imaging, multi-channel techniques, multi-baseline interferometry, and digital beamforming. These innovations aim to extend the swath, improve the SNR, and enable efficient processing while maintaining a high resolution. However, these innovations introduce new challenges, like non-uniform azimuth sampling, necessitating complex interpolation algorithms, and increasing processing complexity.

To address limitations in conventional SAR imaging, this paper proposes a 2D beam scanning SAR to simultaneously improve the swath width and resolution. It mitigates squint angle restrictions on the swath width by incorporating range beam scanning, allowing the maximum width to be defined by the range beam angle. In highly squinted modes where range migration may reduce the scene width, the proposed approach maintains the resolution while reducing the data size, extending the swath, and increasing the azimuth dimension. The beam-steering method performs robustly under varying conditions, ensuring a wide swath, regardless of squint. This is achieved by synergistically combining azimuth and range beam steering rates with careful squint angle consideration to optimize the resolution and coverage.

**Figure 6:**
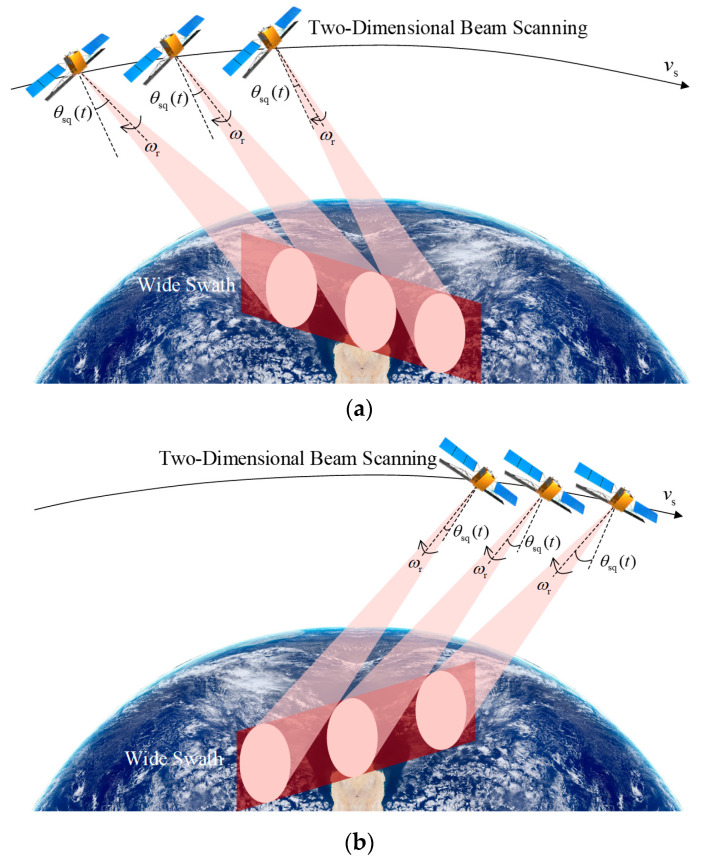
Schematic diagram of the 2D beam scanning SAR. (**a**) Fore-squint wide swath mapping. (**b**) Aft-squint wide swath mapping.

Figure 6 shows different 2D beam scanning patterns between the fore-squint (Figure 6a) and aft-squint (Figure 6b) imaging areas, which both shift from the nadir point. In the fore-squint mode, the beam steering direction aligns with the flight path, while in the aft-squint mode, it orients opposite to the flight direction. The obliquely oriented imaging area with respect to the satellite nadir track enables an increased swath width and extended mapping coverage, with target points distributed non-parallel to the satellite ground track.

**Figure 7:**
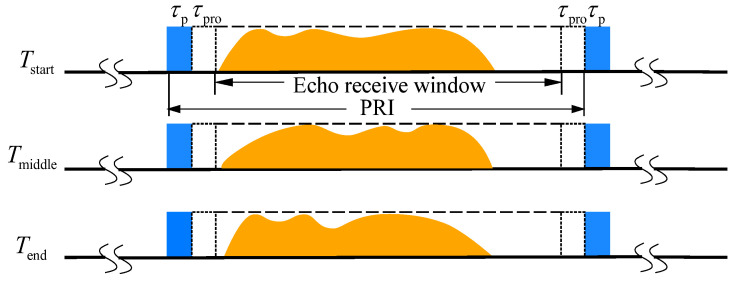
The variation of the 2D beam scanning SAR echo window over time.

As shown in Figure 7, where the echo is represented by the orange area, at squint angles varying from 10° to 20°, the proportions of the echo within the PRI are 64% and 73%, respectively, revealing that throughout the imaging aperture time, the proportion of the echo within the echo window remains large, and no significant range migration occurs.

**Figure 8:**
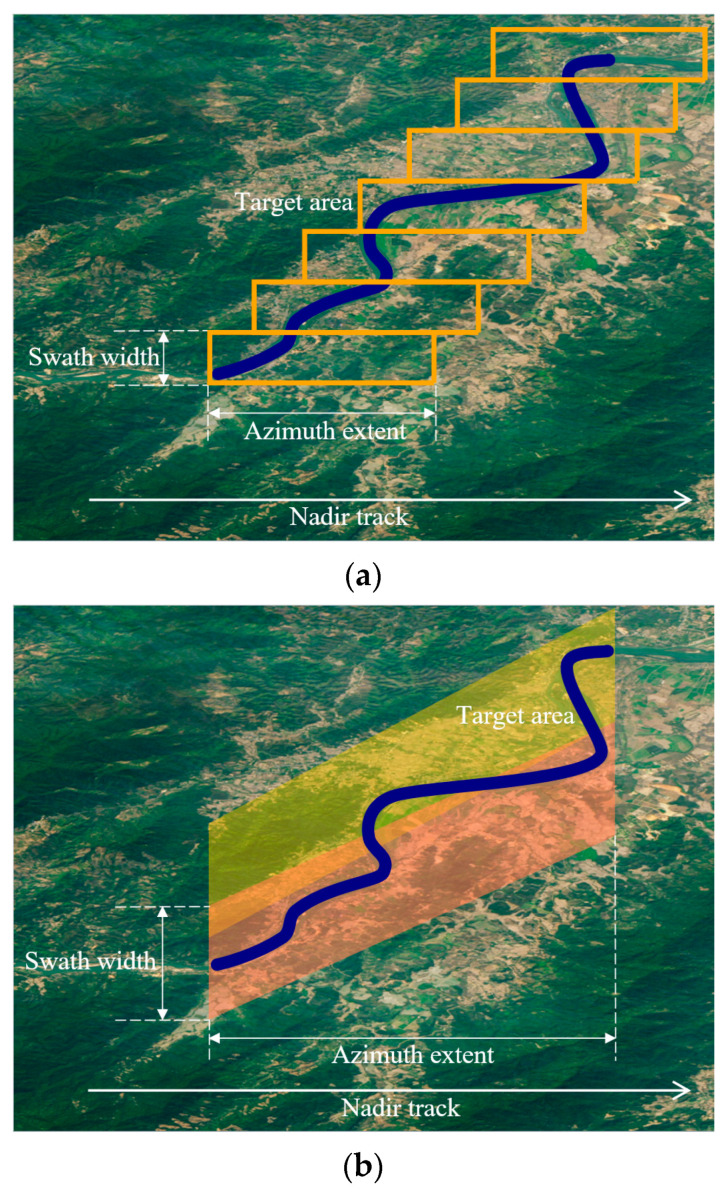
Obliquely oriented imaging area relative to the satellite nadir track, with demonstration of the image-stitching process. (**a**) Traditional squinted SAR. (**b**) Two-dimensional beam scanning SAR.

In addition, the design achieves adaptability to complex observation conditions and ensures imaging accuracy by precisely controlling the echo position in the echo window. This flexibility not only enhances the system’s adaptability but also provides possibilities for future stitching and other advanced applications, as shown in Figure 8. Using squinted 2D beam scanning SAR, only two imaging passes are required to cover the target area, whereas traditional squinted SAR would require seven imaging passes for the same coverage.

### 3.2. Design of Two-Dimensional Beam Scanning

**Figure 9:**
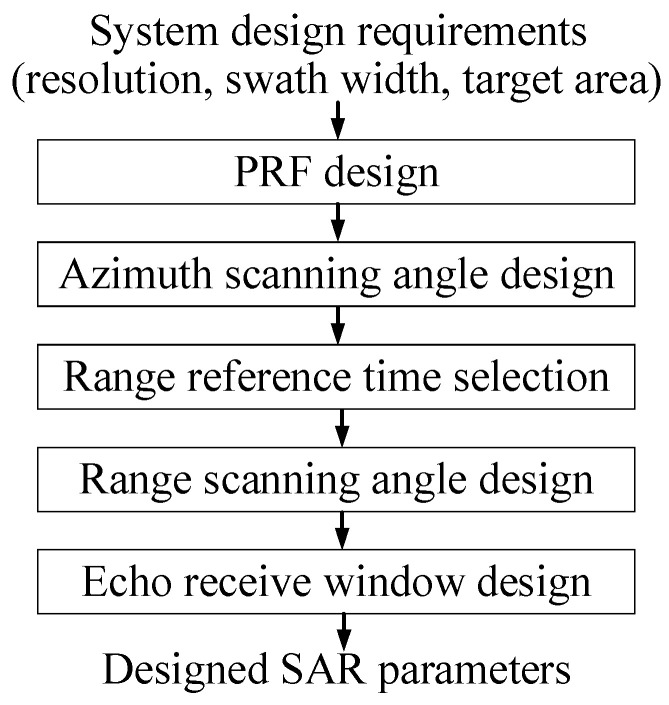
The flow chart of the proposed SAR model system design.

Several pivotal steps comprise the comprehensive design procedure for the proposed 2D beam scanning SAR, as delineated in Figure 9. Initially, the pulse repetition frequency (PRF) is ascertained based on the antenna length and the oversampling rate. Subsequently, the azimuth scanning angle range is tailored to fulfill the azimuth resolution criteria. Thereafter, the optimal reference range time is selected to centralize the echo window. Following this, the range-scanning angular velocity is computed to synchronize with the azimuth beam scanning, thereby maintaining a near-constant squint angle. Lastly, the dimensions of the echo receive window are configured to encompass all echoes across near-to-far ranges. These parameters collectively orchestrate the system’s operating modes and overall performance.

First, the PRF is calculated based on the azimuth antenna length $L_{a}$ and the oversampling rate $\alpha_{s}$, which typically ranges between 1.3 and 1.8 under spaceborne SAR conditions:

(11) $$\mathrm{PRF}=\alpha_{s}\frac{2v_{s}}{L_{a}}$$

where $v_{s}$ represents the radar’s motion along the orbital direction.

The calculation formula for the central squint angle $\theta_{\mathrm{sq},c}$ is as follows:

(12) $$\theta_{\mathrm{sq},c}=\arccos\left(\frac{\Delta y}{R_{c}}\right)$$

where Δy denotes the cross-track distance and $R_{c}$ represents the slant range of the scene center at the central imaging time.

The beam improvement factor *A* in the 2D beam scanning SAR can be calculated with the azimuth resolution $\rho_{a}$ using the following formula:

(13) $$\left\{ \begin{array}{l} A=\frac{L_{a}\cdot \gamma_{\omega ,a}}{2\rho_{a}}\\ \rho_{a}=0.886\frac{\lambda }{2\theta_{\mathrm{sq},c}}\frac{v_{g}}{v_{s}} \end{array}\right.$$

where $\gamma_{\omega ,a}$ represents the azimuth broadening factor introduced by the window function, $L_{a}$ is the length of the azimuth antenna, and $v_{g}$ represents the movement speed of the antenna beam’s projection on the ground.

Based on the given conditions, the azimuth beam scanning angular velocity $\omega_{a}$ can be determined during the imaging process:

(14) $$\omega_{a}=\frac{1-A}{v_{g}\cdot \cos^{2}\theta_{\mathrm{sq},c}}$$

The radar operation time T for the 2D beam scanning SAR can be expressed as follows:

(15) $$T=\frac{L_{\mathrm{sence}}+R_{c}\cdot \theta_{a}}{A\cdot v_{g}}$$

where $L_{\mathrm{sence}}$ denotes the scene length in the azimuth direction ($L_{\mathrm{sence}}=\rho_{a}\cdot A\cdot \omega_{a}$).

The formulas for calculating the squint angle at the starting moment $\theta_{\mathrm{sq},\mathrm{st}}$ and the ending moment $\theta_{\mathrm{sq},\mathrm{end}}$ are as follows:

(16) $$\left\{ \begin{array}{l} \theta_{\mathrm{sq},\mathrm{st}}=\theta_{\mathrm{sq},c}-\frac{\omega_{a}T}{2}\\ \theta_{\mathrm{sq},\mathrm{end}}=\theta_{\mathrm{sq},c}+\frac{\omega_{a}T}{2} \end{array}\right.$$

The calculation formulas for the look angles $\gamma_{\mathrm{eq}}$ at the starting and ending moments are as follows:

(17) $$\left\{ \begin{array}{l} \gamma_{\mathrm{eq},\mathrm{st}}(\gamma ,\theta_{\mathrm{sq},\mathrm{st}};\theta_{\mathrm{az}})=\cos^{-1}\left[\cos\gamma_{c}\cos\left(\theta_{\mathrm{sq},\mathrm{st}}+\theta_{\mathrm{az}}\right)\right]\\ \gamma_{\mathrm{eq},\mathrm{en}}(\gamma ,\theta_{\mathrm{sq},\mathrm{end}};\theta_{\mathrm{az}})=\cos^{-1}\left[\cos\gamma_{c}\cos\left(\theta_{\mathrm{sq},\mathrm{end}}+\theta_{\mathrm{az}}\right)\right] \end{array}\right.$$

Here, $\gamma_{c}$ represents the nadir angle at the central moment ($\gamma_{c}=\arccos(H/R_{c})$).

Based on the azimuth beam scanning angular velocity $\omega_{a}$, the range beam scanning angular velocity $\omega_{r}$ in the 2D beam scanning SAR can be calculated as follows:

(18) $$\omega_{r}=\frac{\gamma_{\mathrm{sq},\mathrm{end}}-\gamma_{\mathrm{sq},\mathrm{st}}}{T}=\frac{(\gamma_{\mathrm{sq},\mathrm{end}}-\gamma_{\mathrm{sq},\mathrm{st}})\cdot A\cdot v_{g}}{\rho_{a}\cdot A\cdot \omega_{a}+R_{c}\cdot \theta_{a}}$$

From the equation, it can be deduced that the $\omega_{r}$ of the 2D beam scanning SAR varies in conjunction with the $\omega_{a}$.

In the context of the 2D beam scanning SAR, the incidence angle $\theta_{\mathrm{inc}}$ can be used to calculate the geocentric angle β:

(19) $$\beta (\gamma ,\theta_{\mathrm{sq}};\theta_{\mathrm{az}})=\theta_{\mathrm{inc}}(\gamma ,\theta_{\mathrm{sq}};\theta_{\mathrm{az}})+\theta_{\mathrm{az}}-\gamma_{\mathrm{eq}}(\gamma ,\theta_{\mathrm{sq}};\theta_{\mathrm{az}})$$

Based on the near-range slant distance $R_{\mathrm{near}}$ and the far-range slant distance $R_{\mathrm{far}}$, the echo window length Δτ can be computed:

(20) $$\Delta \tau =R_{\mathrm{far}}\left(\gamma ,\theta_{\mathrm{sq},\mathrm{end}};\theta_{\mathrm{az}}\right)-R_{\mathrm{near}}\left(\gamma ,\theta_{\mathrm{sq},\mathrm{st}};\theta_{\mathrm{az}}\right)$$

The observation slant angle α, which refers to the angle between the imaging area orientation and the satellite nadir track, is connected with other parameters via the refined formula:

(21) $$\alpha (\gamma ,\theta_{\mathrm{sq}};t)=\arcsin\left(\frac{\sin\theta_{\mathrm{sq}}}{\sin\gamma }\right)$$

By substituting the configured parameter values into the observation slant angle formula, $\alpha_{\mathrm{st}}$ and $\alpha_{\mathrm{end}}$ can be derived as follows:

(22) $$\left\{ \begin{array}{l} \alpha_{\mathrm{st}}(\gamma ,\theta_{\mathrm{sq}};t)=\arcsin\left(\frac{\sin\theta_{\mathrm{sq},\mathrm{st}}}{\sin\gamma_{\mathrm{st}}}\right)\\ \alpha_{\mathrm{end}}(\gamma ,\theta_{\mathrm{sq}};t)=\arcsin\left(\frac{\sin\theta_{\mathrm{sq},\mathrm{end}}}{\sin\gamma_{\mathrm{end}}}\right) \end{array}\right.$$

Thus, the range of the observation slant angle α in the 2D beam scanning SAR is $\alpha \in \left[\alpha_{\mathrm{st}},\alpha_{\mathrm{end}}\right]$. Therefore, based on the system parameters listed in Table 1, by controlling the observation slant angle α to vary between 23.5° and 25.5° in the design of the 2D beam scanning SAR, a flexible approach is employed to keep the slant range largely unchanged, while permitting minor variations within a small range.

**Figure 10:**
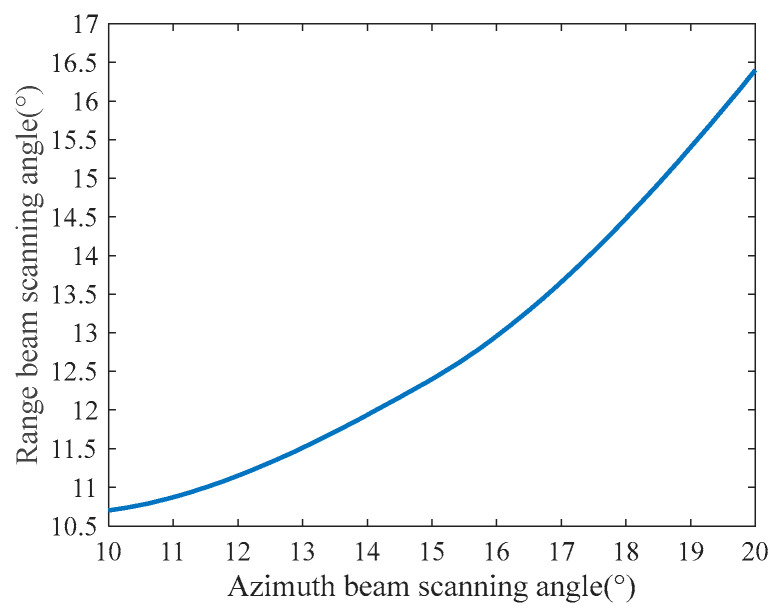
Imaging area of the 2D beam scanning mode with a central squint angle of 15°.

Figure 10 demonstrates the cooperative control of azimuth and range beam scanning angles for 2D beam scanning SAR. With a 15° central squint, the azimuth beam scans ±5° while the range beam varies from 10.7° to 16.4°, enabling flexible 2D steering. This joint angle control ensures efficient echo utilization for high-resolution, wide-swath imaging.

**Figure 11:**
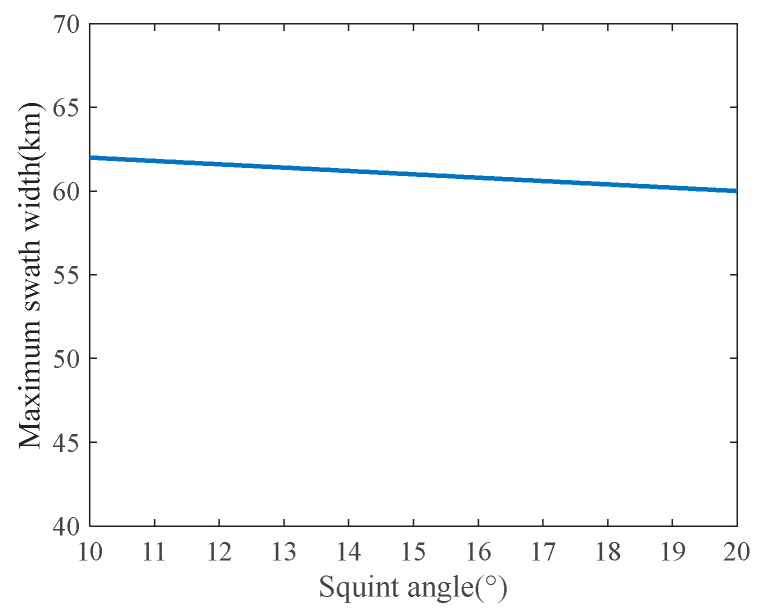
Mapping bandwidth achievable with the 2D beam scanning SAR.

Based on the system parameters, the derived slant ranges are 581 km at the start and 663 km at the scene center. Figure 11 presents the maximum swath width under this mode, showing a scene width of approximately 60 km at different squint angles. The coordinated azimuth and range beam scanning facilitates wide-area imaging by harnessing multi-angle observations in both dimensions.

## 4. Imaging Algorithm Analysis

In this study, two critical issues were addressed in the preprocessing of 2D beam scanning SAR data. The first issue arises from the fact that the imaging area in 2D beam scanning SAR is not parallel to the satellite’s nadir track and has a relatively large swath width. This leads to an insufficient number of sampling points in the range direction. The second issue is inherent to sliding spotlight SAR, which already has a sampling rate and a long imaging time. When squint is introduced, the problem of Doppler spectrum aliasing becomes severe. To address these challenges, a two-step upsampling azimuthal deskewing preprocessing method is proposed, which is structured into four major steps. The first step involves range zero padding to augment the number of sampling points in the range direction. The subsequent three steps are dedicated to azimuthal upsampling, aiming to alleviate the Doppler aliasing issue and to ensure a uniform azimuthal sampling rate.

**Figure 12:**
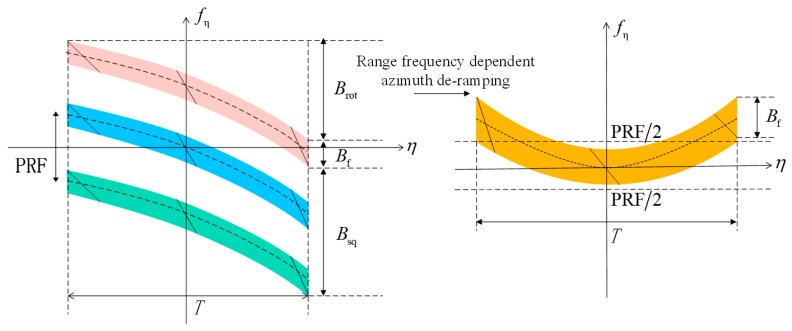
Azimuth time–frequency plot post-deskewing.

As is evident from Figure 12, the additional squint leads to substantial extra bandwidth, as well as long temporal scan angles. This results in Doppler spectra aliasing. After distance–frequency-dependent de-ramping, the spectra continue to exhibit aliasing. In this context, different colored regions within the spectra represent data blocks with diverse PRF. The azimuthal total bandwidth for 2D beam scanning SAR data is restricted within the azimuthal sampling frequencies designated for these individual data blocks. However, a segment does surpass these predefined limits. Frequency-dependent de-ramping also has its limitations. To enable smooth concatenation of azimuthal data blocks in later processing, an azimuthal resampling procedure is essential to transform the non-uniformly sampled azimuth blocks into a uniformly sampled signal structure [16].

**Figure 13:**
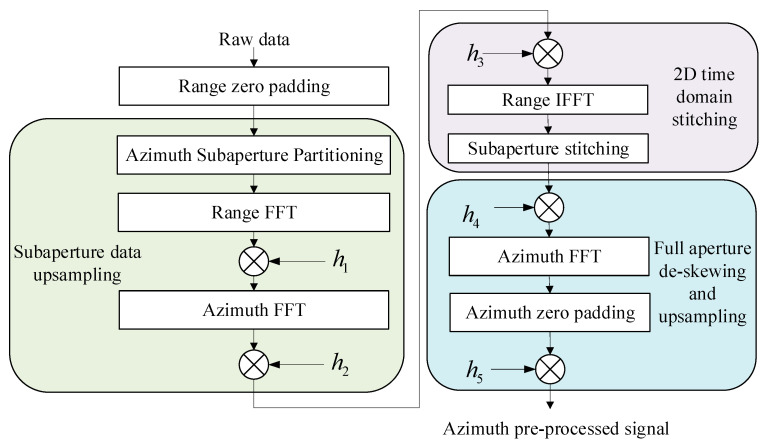
Flowchart illustrating the preprocessing procedure for distance–frequency-dependent de-ramping.

As illustrated in Figure 13. First, range zero padding is performed to provide an increased number of sampling points. In the context of 2D beam scanning SAR, the introduction of range beam scanning angles can result in a misalignment between the data points and their corresponding positions, which is primarily attributed to the system operating with a unified Doppler center. To address this issue, it is essential to accurately calculate the additional number of range samples via zero padding to realign the range data points with their true spatial positions:

(23) $$N_{\mathrm{add}}\geq \left[\left\{\frac{2\left[R(\gamma_{\mathrm{far}},\theta_{\mathrm{sq}};\theta_{a}/2)-R(\gamma_{\mathrm{far}},\theta_{\mathrm{sq}};-\theta_{a}/2)\right]}{c}\right\}+\tau_{p}\right]\cdot f_{s}-N_{r}$$

where $N_{\mathrm{add}}$ represents the additional number of range samples, $N_{r}$ represents the original number of range samples and $f_{s}$ denotes the sampling frequency.

In the second stage of the algorithm, upsampling of the subaperture data is performed. This is followed by the application of a frequency-dependent deskewing technique. The adoption of this specific deskewing approach is motivated by the need to mitigate the effects of the additional bandwidth introduced under high squint angles. $h_{1}\left(f_{\tau },i\cdot \Delta \eta \right)$ is employed to achieve frequency-dependent deskewing through convolution. The FFT is utilized to expedite the computational process. It is worth noting that all azimuthal operations are conducted in the time domain, and at this stage, no range cell migration correction is applied.

(24) $$h_{1}\left(f_{\tau },i\cdot \Delta \eta \right)=\exp\left\{-j\pi \frac{2\cdot v_{s}\cdot (f_{c}+f_{\tau })\cdot \omega_{r}}{c}{\left(i\cdot \Delta \eta \right)}^{2}\right\}$$

where Δη=1/PRF, $i=-N_{r}/2,\;\ldots ,\;N_{r}/2-1$, and $f_{c}$ represents the carrier frequency. To recover the original signal without aliasing, azimuth upsampling should be executed in the azimuth frequency domain via azimuth zero padding. Consequently, the number of azimuth samples is updated after zero padding.

(25) $$P=N_{r}+\left[k\left(\frac{v_{s}\cdot \theta_{\max}\cdot B_{r}}{c}+\frac{2v_{s}(f_{c}+B_{r}/2)}{c}\cdot \theta_{a}\right)-\mathrm{PRF}\right]\cdot T_{\mathrm{sub}}$$

where k generally ranges between 1.2 and 1.5, with a chosen value of 1.2 for this case; $\theta_{\max}$ denotes the maximum azimuth beam scanning angle

(26) $$h_{2}\left(f_{\tau },p\cdot \Delta \eta_{1}\right)=\exp\left\{-j\pi \frac{2\cdot v_{s}\cdot (f_{c}+f_{\tau })\cdot \omega_{r}}{c}{\left(p\cdot \Delta \eta_{1}\right)}^{2}\right\}$$

where $\Delta \eta_{1}={1/\mathrm{PRF}}_{1}$, ${\mathrm{PRF}}_{1}=P/p\cdot T$, and p=−P/2, …, P/2.

In the third phase of the algorithm, full-aperture deskewing and upsampling are executed. Unlike the previous step, the deskewing process employed here is not distance–frequency dependent; it is solely a deskewing operation. This stage necessitates a high sampling rate due to the substantial bandwidth retained post-deskewing. The requirements for a high sampling rate are met at this point, thanks to the upsampling procedure carried out in the preceding stage. Incorporated within this third phase is the function $h_{3}\left(f_{\tau },i\Delta \eta_{1}\right)$, which serves to perform inverse deskewing.

(27) $$h_{3}\left(f_{\tau },i\cdot \Delta \eta_{1}\right)=\exp\left\{j\pi \frac{2\cdot v_{s}\cdot (f_{c}+f_{\tau })\cdot \omega_{r}}{c}{\left(i\cdot \Delta \eta_{1}\right)}^{2}\right\}$$

In the final stage of our algorithm, a comprehensive procedure termed full aperture deskewing and upsampling is executed. This involves a sequence of steps, specifically, $h_{4}\left(\tau ,p\cdot \Delta \eta_{1}\right)$ is defined as

(28) $$h_{4}\left(\tau ,p\cdot \Delta \eta_{1}\right)=\exp\left[-j\pi \frac{2v_{s}\omega_{r}f_{c}}{c}{\left(p\cdot \Delta \eta_{1}\right)}^{2}\right]$$

and $h_{5}\left(\tau ,q\cdot \Delta \eta_{1}\right)$ is given by

(29) $$h_{5}\left(\tau ,q\cdot \Delta \eta_{2}\right)=\exp\left[-j\pi \frac{2v_{s}\omega_{r}f_{c}}{c}{\left(q\left|\frac{cP}{2v_{s}\omega_{r}f_{c}TQ}\right|\right)}^{2}\right]$$

where $\Delta \eta_{2}=1/{\mathrm{PRF}}_{2}$, Q represents the total number of points after azimuth zero padding, and q=−Q/2, …, Q/2.

These operations collectively yield the azimuth preprocessed signal, effectively addressing the challenges associated with azimuthal non-uniformities and ensuring a high-quality imaging result.

**Figure 14:**
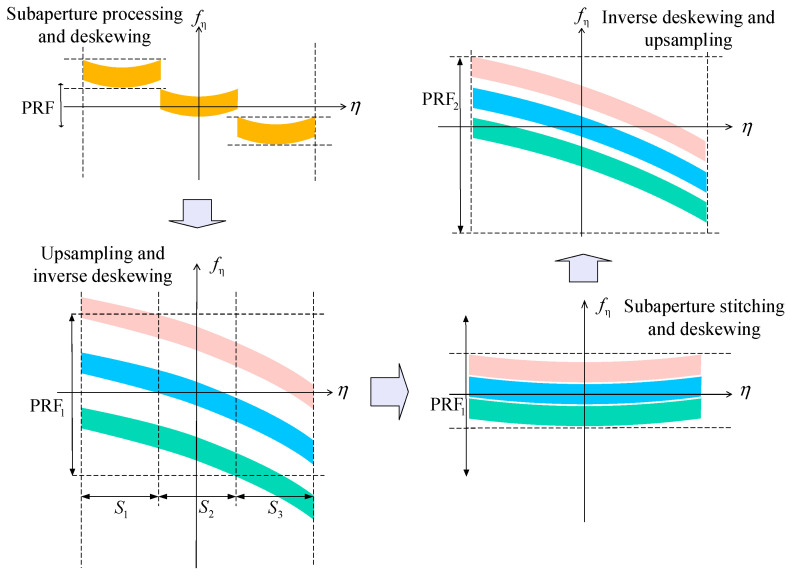
Azimuth time–frequency diagrams after different post-deskewing plots.

As depicted in the first subfigure of Figure 14, subaperture partitioning and deskewing are performed on the azimuth time–frequency diagram. The initial deskewing is a range frequency-dependent operation, with identical deskewing applied to each range gate. Subaperture partitioning aims to limit the bandwidth within each subaperture below the PRF, avoiding aliasing. After this frequency-dependent deskewing, frequency variations are effectively normalized, yielding a uniform spectral distribution in the azimuth direction for each subaperture.

In the subsequent subfigure, the subaperture data undergo upsampling and inverse deskewing. This ensures that each segmented subaperture maintains an adequate sampling rate.

In the third subfigure, subaperture stitching and deskewing are executed as part of the full-aperture processing. This deskewing operation is conducted in both the azimuth and range time domains. Notably, each distinct range gate undergoes a unique deskewing process. The objective of this step is to seamlessly concatenate the subapertures, thereby achieving full-aperture coverage. This deskewing is distance dependent and tailored to the specific characteristics of each range gate.

Subsequently, inverse deskewing and upsampling are performed to finalize the data processing. This ensures that the full-aperture data is uniformly sampled and ready for subsequent imaging steps.

## 5. Simulation Results

In this section, the imaging performance of the 2D beam scanning SAR was validated via simulations. According to Table 1, the obtained mapping swath width was 60 km, the squint angle ranged from 20° to 10°, the azimuth coverage length was 134 km, the initial look angle ranged from 18° to 25°, and the azimuth resolution was 0.5 m.

**Figure 15:**
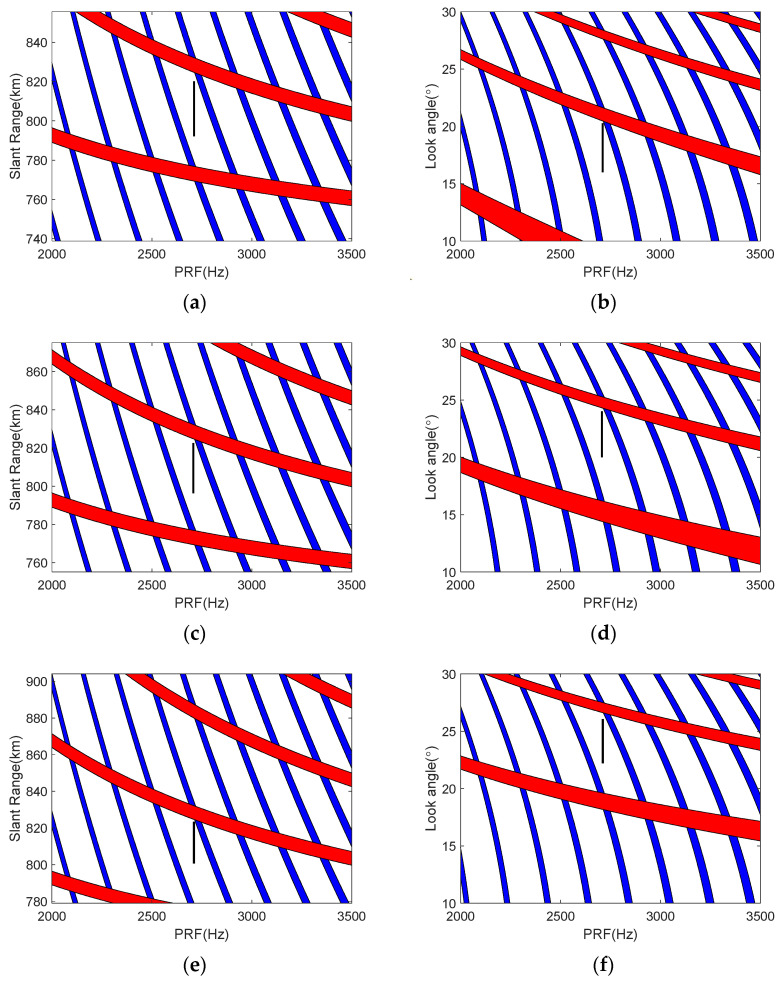
Timing diagram showing selected ranges at different times. (**a**,**b**) Initial moment. (**c**,**d**) Central moment. (**e**,**f**) Ending moment.

Figure 15 demonstrates the robustness of the 2D beam scanning mode, the black line area indicates the range of selected PRF values. Specifically, the PRF was maintained at 2700 Hz. The substantial squint angle change from 20° to 10° resulted in considerable look angle variations from 18° to 25°. Despite these squint-angle-induced look angle changes, the slant range exhibited minimal fluctuations across different moments. This indicates the echo window position remained relatively stable amidst significant look angle variations caused by the squint angle changes. The stability in slant range and echo window amidst the considerable squint and resultant look angle changes, highlights the capability of 2D beam scanning to maintain imaging consistency.

**Figure 16:**
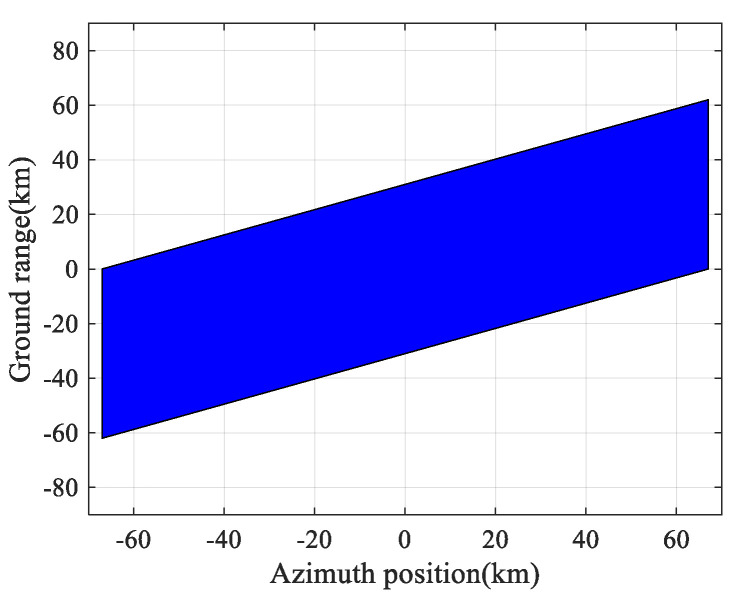
Imaging area of the 2D beam scanning SAR with a central squint angle of 15°.

As shown in Figure 16, the 2D beam scanning SAR’s imaging area was not aligned with the satellite’s nadir track. The swath width remained relatively stable, maintaining a range of around 60 km, while the azimuthal imaging length extended to 134 km.

**Figure 17:**
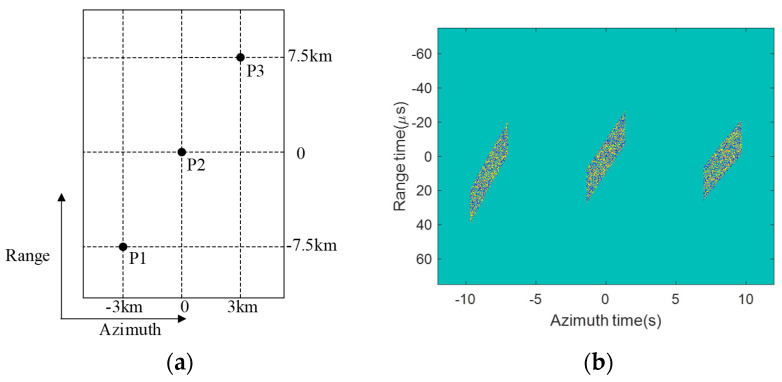
Point target scene and echo data. (**a**) Distribution of target in the scene. (**b**) Real part of echo data.

As shown in Figure 17a, despite point P3 being at a farther slant range, its echo in Figure 17b is located at almost the same range position as P1 and P2. This is attributed to the smaller squint angle of 14.6° for P3 compared with 15.4° for P1 and 15° for P2. The comparable echo ranges for points at different slant ranges further validate the effectiveness of coordinated control of squint and look angles in suppressing range cell migration in the proposed 2D beam scanning SAR.

**Figure 18:**
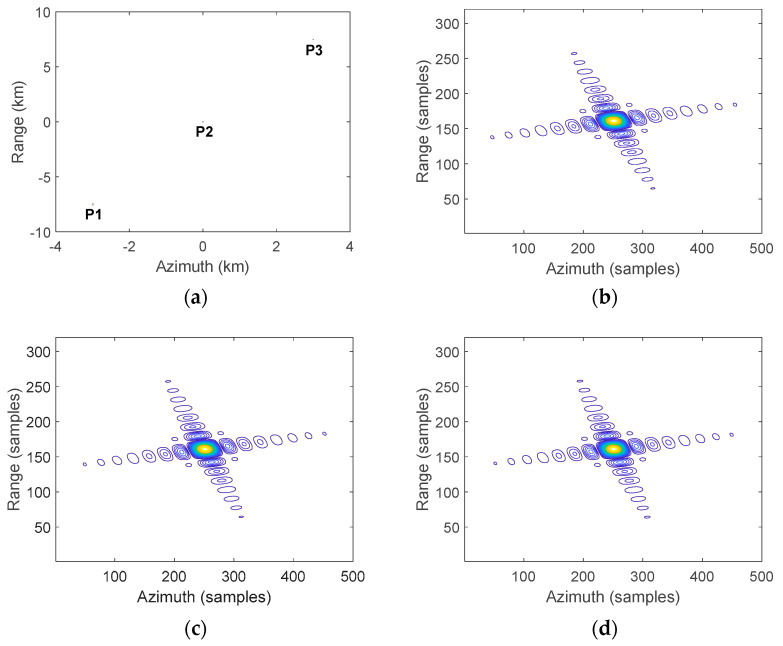
Imaging outcomes for three point targets processed using the suggested approach. (**a**) Imaging results with three points. (**b**) Contour plot of P1. (**c**) Contour plot of P2. (**d**) Contour plot of P3.

Figure 18 shows the imaging results of three point targets P1, P2, and P3, with their contour lines demonstrating effective focusing performance. It can be observed that each target point was well focused into a tight impulse response with the proposed 2D beam scanning SAR approach. This provides a validation of the efficacy of the proposed approach for high-resolution focusing over a wide swath.

**Figure 19:**
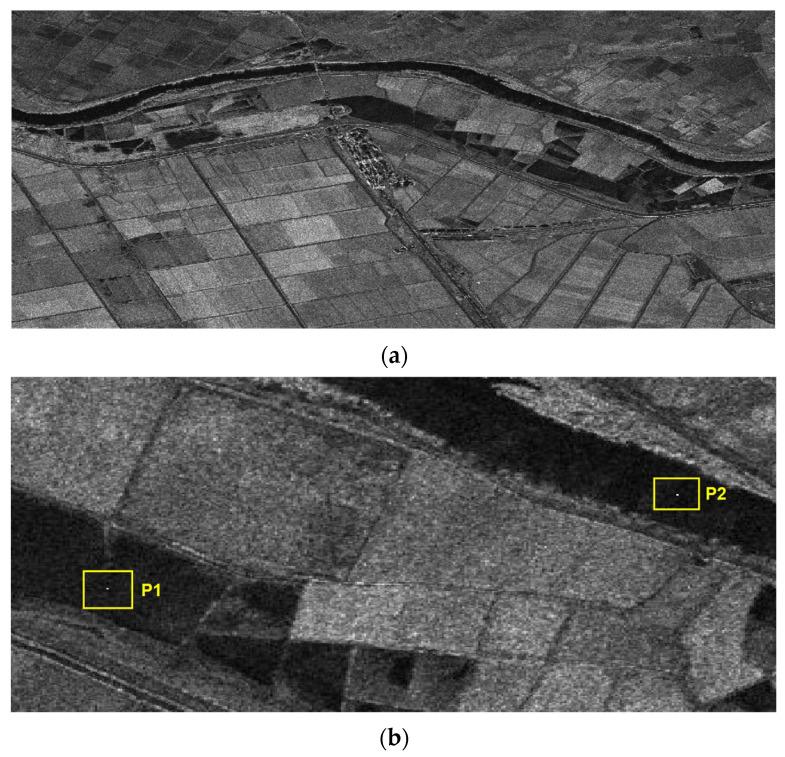
Imaging simulation scene. (**a**) Full imaging scene. (**b**) Zoomed-in view of point targets.

In order to further verify the superiority of the proposed 2D beam scanning mode, Figure 19a presents the complete imaging scene covering the coastal region along the Greece–Turkey border, including complex terrain across the Ipsala and Edirne area. Figure 19b provides a zoomed-in view of two bright point targets P1 and P2, which were selected for examining the focusing performance.

**Figure 20:**
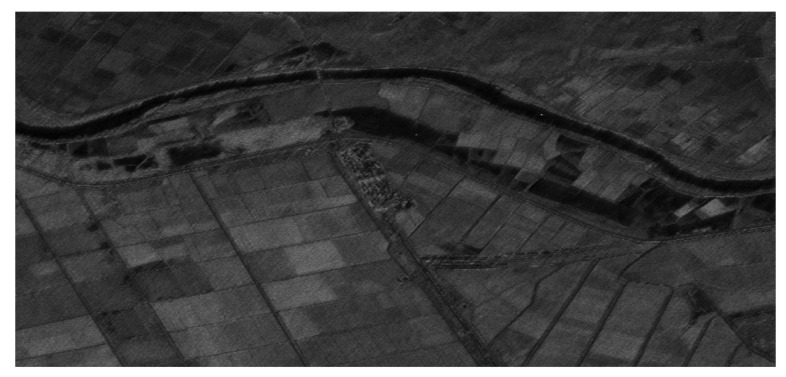
Processed results of distributed targets.

Figure 20 shows the processed results of distributed targets covering the complete wide swath area of interest after applying the proposed preprocessing procedures. The oblique imaging scene along the Greece–Turkey border was well reconstructed, without noticeable artifacts from the preprocessing steps. The varying reflectivity of the distributed targets across the complex landscape around Ipsala and Edirne can be clearly observed in Figure 20. This validates the efficacy of the proposed 2D beam scanning SAR system in accurately imaging distributed scatterers over large areas. Overall, the good focusing and reconstruction of distributed scenes validated the effectiveness of the proposed approach with joint optimization across multiple domains.

**Figure 21:**
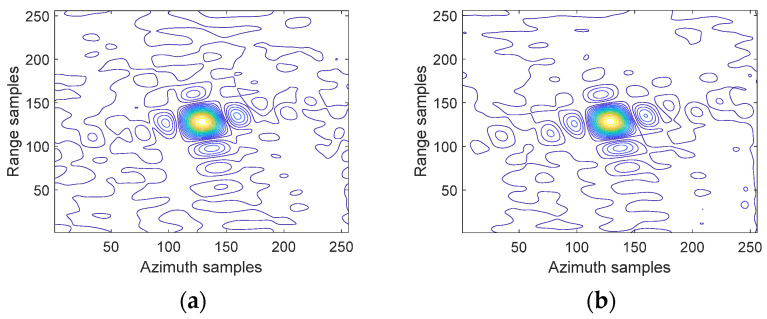
Focusing results of point targets. (**a**) Contour plot of P1. (**b**) Contour plot of P2.

As exhibited in Figure 21a,b, point targets P1 and P2 were well focused into sharp impulse responses with narrow peak widths, demonstrating the excellent focusing capability of the 2D beam scanning mode across the wide image swath. In these point target images, different colors are used to represent different signal intensities, providing a more intuitive understanding of the reflective characteristics of the targets. The impulse response peak of P1 was confined within an area of 0.52 m × 0.52 m, achieving an azimuth resolution of 0.52 m. Similarly, the impulse response peak of P2 was limited to an area of 0.51 m × 0.51 m, reaching an azimuth resolution of 0.51 m. These quantified resolution results validated that the proposed 2D beam scanning approach realizes sub-meter resolution for point targets across the 60 km swath width, meeting the high-resolution requirement.

## 6. Conclusions

This paper proposes a novel 2D beam scanning technique for squinted sliding spotlight SAR imaging. Using synchronous steering of azimuth and range beams, it achieves wider swath coverage and flexible imaging geometry while suppressing range cell migration. Specifically, cooperative azimuth-range beam adjustment maintains stable equivalent nadir angles for reduced migration over large squint angles. Meanwhile, bidirectional beam scanning acquires richer spatial sampling without an extra data load. These advantages enable focused wide-swath SAR imaging with high resolution, which is suitable for efficient large-area mapping and monitoring. The signal models and preprocessing algorithms presented also accurately reconstruct the azimuth signal history and Doppler parameters for effective motion compensation and image focusing. It is acknowledged that more advanced antenna systems and beamforming networks capable of flexible 2D beam steering need to be developed to implement the proposed technique, which increases the hardware complexity compared with conventional spotlight SAR. In summary, the proposed 2D scanning SAR provides an innovative solution to overcome limitations in conventional scanning modes, with promising applications in disaster monitoring, resource mapping, and environmental surveillance. Future research will focus on extending this technique to highly nonlinear orbits and developing new algorithms to further exploit its potential.

## Figures and Tables

**Table 1:** Simulation parameters.

Parameter	Value
Orbital height	714 km
Earth’s radius	6371 km
Wavelength	0.03125
Azimuth antenna length	7 m
Pulse width	20 μs
Platform velocity	7560 m/s
Equivalent velocity	7250 m/s

## Data Availability

Not applicable.

## References

[1] Shen S., Nie X., Zhang X. (2018). Research on Synthetic Aperture Radar Processing for the Spaceborne Sliding Spotlight Mode. Sensors.

[2] Chen J., Kuang H., Yang W., Liu W., Wang P. (2016). A Novel Imaging Algorithm for Focusing High-Resolution Spaceborne SAR Data in Squinted Sliding-Spotlight Mode. Remote Sens. Lett..

[3] Gao Z., Wei C., Yang C., Xie Y., Chen H. (2019). Parallel processing of sliding spotlight mode SAR imaging based on GPU. J. Eng..

[4] Sun Z., Chen T., Sun H., Wu J., Lu Z., Li Z., An H., Yang J. (2022). A Novel Frequency-Domain Focusing Method for Geosynchronous Low-Earth-Orbit Bistatic SAR in Sliding-Spotlight Mode. Remote Sens..

[5] Awada E., Radwan E., Nour M. (2022). Robust sliding mode controller for buck DC converter in off-grid applications. Bull. Electr. Eng. Inform..

[6] Zhang Q., Xiao F., Ding Z., Ke M., Zeng T. (2018). Sliding Spotlight Mode Imaging with GF-3 Spaceborne SAR Sensor. Sensors.

[7] Yang W., Chen J., Zeng H., Zhou J., Wang P., Li C. (2012). A Novel Three-Step Image Formation Scheme for Unified Focusing on Spaceborne Sar Data. Prog. Electromagn. Res..

[8] Ge N., Rodríguez González F., Wang Y., Shi Y., Zhu X. (2018). Spaceborne Staring Spotlight SAR Tomography—A First Demonstration with TerraSAR-X. IEEE J. Sel. Top. Appl. Earth Obs. Remote Sens..

[9] Zheng Y., Yi W., Guan J., Ye X., Tao S. (2022). An Improved Equivalent Acceleration Imaging Approach for Spaceborne Sliding Spotlight SAR. AIP Adv..

[10] Liu S., Kong W., Chen X., Xu M., Yasir M., Zhao L., Li J. (2022). Multi-Scale Ship Detection Algorithm Based on a Lightweight Neural Network for Spaceborne SAR Images. Remote Sens..

[11] Men Z., Wang P., Li C., Chen J., Liu W., Fang Y. (2017). High-Temporal-Resolution High-Spatial-Resolution Spaceborne SAR Based on Continuously Varying PRF. Sensors.

[12] Xu W., Zhang Z., Huang P., Tan W., Qi Y. (2023). System Design and Signal Processing in Spaceborne Squint Sliding Spotlight SAR with Sub-Aperture Block-Varying PRF. Electronics.

[13] Li Y., Wu H., Meng D., Gao G., Lian C., Wang X. (2022). Ground Positioning Method of Spaceborne SAR High-Resolution Sliding-Spot Mode Based on Antenna Pointing Vector. Remote Sens..

[14] Kim H., Park J., Chang Y.-K., Lee S.-H. (2021). Optimal Attitude Maneuvering Analyses for Imaging at Squint Staring and Sliding Spotlight Modes of SAR Satellite. Aerospace.

[15] Hu X., Wang P., Zeng H., Guo Y. (2021). An Improved Equivalent Squint Range Model and Imaging Approach for Sliding Spotlight SAR Based on Highly Elliptical Orbit. Remote Sens..

[16] Zhang Z., Xu W., Huang P., Tan W., Gao Z., Qi Y. (2022). Azimuth Full-Aperture Processing of Spaceborne Squint SAR Data with Block Varying PRF. Sensors.

[17] Wang Y., Yang J., Li J. Data acquisition for a novel spaceborne azimuth-range sweep synthetic aperture radar. Proceedings of the IEEE International Geoscience and Remote Sensing Symposium.

[18] Wang Y., Li J., Yang J. (2017). Wide Nonlinear Chirp Scaling Algorithm for Spaceborne Stripmap Range Sweep SAR Imaging. IEEE Trans. Geosci. Remote Sens..

[19] Xu W., Li R., Fang C., Huang P., Tan W., Qi Y. (2021). Azimuth Multichannel Reconstruction Based on Advanced Hyperbolic Range Equation. Remote Sens..

[20] Alshaya M., Yaghoobi M., Mulgrew B. (2020). High-Resolution Wide-Swath IRCI-Free MIMO SAR. IEEE Trans. Geosci. Remote Sens..

[21] Guccione P., Mapelli D., Giudici D., Persico A.R. (2022). Design of f-SCAN Acquisition Mode for Synthetic Aperture Radar. Remote Sens..

[22] Mou J., Wang Y., Hong J., Wang Y., Wang A. (2023). Baseline Calibration of L-Band Spaceborne Bistatic SAR TwinSAR-L for DEM Generation. Remote Sens..

[23] Gebert N., Villano M., Krieger G., Moreira A. (2013). Digital Beamforming on Receive: Techniques and Optimization Strategies for High-Resolution Wide-Swath SAR Imaging. IEEE Trans..

